# Metronomic oral cyclophosphamide (MOC) in the salvage therapy of heavily treated recurrent ovarian cancer patients: a retrospective, multicenter study

**DOI:** 10.1186/1471-2407-14-947

**Published:** 2014-12-13

**Authors:** Gabriella Ferrandina, Giacomo Corrado, Floriana Mascilini, Paola Malaguti, Riccardo Samaritani, Mariagrazia Distefano, Valeria Masciullo, Alessia Di Legge, Antonella Savarese, Giovanni Scambia

**Affiliations:** Gynecologic Oncology Unit, Catholic University of Rome, Rome, Italy; Gynecologic Oncology Unit, Catholic University of Campobasso, Campobasso, Italy; Department of Medical Oncology, “Regina Elena” National Cancer Institute, Rome, Italy; Medical Oncology Unit, “Regina Margherita” Hospital, Rome, Italy; Department of Surgical Oncology, Gynecologic Oncology Unit “Regina Elena” National Cancer Institute, IFO - Via Elio Chianesi 53-00144, Rome, Italy

**Keywords:** Cyclophosphamide, Metronomic administration, Ovarian cancer, Salvage treatment

## Abstract

**Background:**

The aim of this multicenter, retrospective study was to evaluate the efficacy and safety of metronomic oral cyclophosphamide (MOC) in heavily treated, relapsed ovarian cancer (ROC) patients.

**Methods:**

oral cyclophosphamide (Endoxan®, Baxter, Italy) was administered at the dose of 50 mg daily, continuously. Treatment-related toxicity and response to treatment were assessed by the NCI-CTC criteria, and RECIST criteria, respectively. Progression-free (PFS), and overall survival (OS) were also assessed.

**Results:**

54 patients were analyzed: 20 patients (37.0%) were considered primarily platinum refractory/resistant, while 34 patients (63.0%) were defined as platinum sensitive; 79.6% of patients had received ≥2 previous lines before starting MOC. The objective response rate (ORR) was 20.4%. Eleven patients (20.4%) experienced stable disease and 8 of them had a response duration ≥6 months. A total of 32 patients (59.2.%) progressed during treatment. Median PFS was 4 months, and the 12-month PFS rate was 19.6%; median OS was 13 months, and the 12-month OS rate was 51.5% . Patients responding to MOC showed a more favorable PFS (median = 17 months) compared to patients with stabilization (median = 6 months) or progression of disease (median = 3 months) (p value = 0.0001). Median OS of responding patients was 30 months compared to 11 months in cases achieving stabilization, or progression of disease (median = 8 months) (p value = 0.0001). Only 1 patient experienced grade 3 anemia. Non-hematological grade 3 toxicity was registered in 2 patients.

**Conclusions:**

MOC could provide a valid alternative in terms of risk/benefit ratio in the palliative treatment of heavily treated ROC patients.

## Background

Despite the advances in surgical efforts, and the achievement of high response rates with standard platinum/paclitaxel front-line treatment, ovarian cancer remains the most lethal gynaecological malignancy with a 5-year survival rate of 25-30% in advanced stage disease [1, 2]. Major determinants of clinical outcome are represented by the extent of residual tumor at primary surgery, and sensitivity to platinum-based chemotherapy [3, 4]. In particular, patients recurring within 6 months from initial therapy exhibit low rates of response (4-23%) to salvage treatment, and a median overall survival of approximately a year [3]. In this clinical setting, special attention has to be paid to the issue of palliation of symptoms and preservation of quality of life which remain the most realistic objectives. Conversely, patients who recur >12 months after initial therapy are defined as fully platinum sensitive, and are usually re-challenged to platinum based combinations [4], while patients with a platinum free interval within 6–12 months from primary treatment are considered as partially platinum sensitive, and seem to attain rates of response to platinum re-challenge similar to those achieved with non platinum agents [5]. Given the higher chance of response to chemotherapy and the more favorable prognosis compared to platinum refractory/resistant setting, platinum sensitive patients often undergo several lines of treatments with the intent to prolong survival while maintaining an acceptable quality of life.

In this context, besides privileging less toxic drugs, oral administration, and outpatient setting rather than hospital-based therapies, also the frequent or even continuous administration of low dose chemotherapy (i.e. metronomic chemotherapy) has gained much attention in recent years; indeed, this route of drug administration has been shown to be as active and, in some circumstances, even more efficacious than conventionally administered chemotherapy, in spite of a negligible toxicity [6–9]. Antitumor activity induced by metronomic drug administration has been ascribed mainly to its anti-angiogenetic effects [7–10]; however, the documentation that metronomic chemotherapy activates antitumor immunity as well as tumor dormancy and senescence has established metronomic chemotherapy as a sort of multi-targeted treatment [11, 12].

In the scenario of palliative treatments for heavily treated, recurrent ovarian cancer (ROC) patients, much attention has been focused on metronomic oral cyclophosphamide (MOC): in particular, very early experiences had suggested its possible activity in advanced ovarian cancer patients failing treatment or not amenable to surgery or radiotherapy [13]. More recently, anedoctal cases and small sample series have highlighted the promising rates of response to MOC in ROC patients [14–16].

These observations prompted us to evaluate in a retrospective, multicenter study the efficacy as well as toxicity of MOC in a population of very heavily treated ROC patients.

## Methods

### Study design

This is a multicenter, retrospective, uncontrolled study aimed at evaluating the activity of MOC as single agent in heavily treated ROC patients.

Written informed consent to treatment and to use of clinical data for scientific purposes had been provided by all patients at time of chemotherapy administration. Retrospective collection of clinical data from clinical charts was approved by the Catholic University Ethic Committee and Institutional review Board of Rome (RPN A2116).

Clinical data were collected from four Italian Institutions which had employed single agent MOC in relapsed ovarian cancer patients since April 2007 and April 2013.

Consecutive patients with histologically confirmed epithelial ovarian carcinoma, previously treated with at least one platinum/paclitaxel regimen, and with radiological evidence of measurable recurrence/progression of disease were included in the study. Further selection criteria were: age over 18 years, Eastern Cooperative Oncology Group (ECOG) performance status ≤ 2, life expectancy >3 months, absolute neutrophil count (ANC) >1,500/mm^3^; platelets count >150,000/mm^3^; bilirubin and creatinine levels less than 1.5 times the upper limit of normal; normal cardiac function defined as LVEF ≥ 50%. Pre-treatment evaluation included pelvic examination, abdomino-pelvic CT, Ca125 assay.

All patients were prescribed oral cyclophosphamide (Endoxan®, Baxter, Italy) at the dose of 50 mg daily, continuously Treatment cycle was considered as lasting 30 days. Treatment-related toxicity was assessed according to NCI-CTC criteria (version 2.0) [17] for patients completing at least one cycle of therapy.

### Assessment of response and clinical outcome

Response to treatment was classified according to RECIST criteria (version 1.0) [18]. Patients with rapidly progressive disease, early death from malignant disease or unknown response because of insufficient or unknown data were considered as failing to respond to treatment (progression of disease). Response was also evaluated according to Ca125 levels (GCIG criteria) [19]. Progression-free (PFS), and overall survival (OS) were also assessed.

### Statistical analysis

The χ2 test or Fisher’s exact test for proportion or Mann–Whitney non parametric test were used to analyze the distribution of categorical or continuous data between groups.

Objective response rate (ORR) included complete and partial response. Clinical benefit included complete, partial response, and stabilization of disease. The 95% confidence intervals (95% CI), have been provided. PFS was defined as the time elapsed between start of treatment and documentation of progressive disease or the date last seen; OS was defined as time elapsed between start of CTX and date of death or the date of last follow-up. Medians and life tables were computed using the product-limit estimate by the Kaplan and Meier method [20] and the log-rank test was employed to assess the statistical significance [21]. Statistical analysis was carried out using SOLO (BMDP Statistical Software, Los Angeles, CA, USA).

## Results

### Patient characteristics

A total of 54 patients were analyzed: at initial diagnosis, most patients (83.3%) had serous ovarian carcinoma, and presented with FIGO stage IIIC disease (n = 45, 83.3%) (data not shown). Median age at diagnosis of recurrence was 69 years (range = 40-89). Median PFI was 7.5 months (range = 1-72); 20 patients (37.0%) were considered primarily platinum refractory/resistant, while 34 patients (63.0%) were defined as platinum sensitive (Table 1).

**Table 1:** **Clinico-pathological features of patients at time of MOC administration**

	All cases (N = 54)
**Age at diagnosis of recurrence, yrs**	
Median (range)	69 (40–89)
ECOG PS	
0	20 (37.0)
1	18 (33.3)
2	16 (29.6)
Platinum sensitivity	
Refractory/resistant	20 (37.0)
Sensitive	34 (63.0)
No. prior regimens	
1-2	11 (20.4)
3-4	23 (42.6)
5-6	16 (29.6)
≥7	4 ( 7.4)
Previous anthracyclines	
No	14 (25.9)
Yes	40 (74.1)
Pattern of recurrence	
Carcinomatosis only	9 (16.7)
Carcinomatosis and parenchymal metastasis	8 (14.8)
Carcinomatosis and lymph node disease	14 (25.9)
Carcinomatosis, parenchymal metastases and lymph node disease	12 (22.2)
Isolated parenchymal metastases or lymph node disease	11 (20.4)
Ca125 levels (IU/ml) at baseline	
Median (range)	508 (10–9,500)
≤35 I.U./ml	3 ( 5.6)
>35 I.U./ml	51 (94.4)

Median number of previous regimens was 4 (range = 1-9); in particular, 79.6% of patients had received ≥2 previous lines, and 37.0% of patients had received ≥5 prior treatments before starting MOC. The vast majority of patients (N = 40, 74.1%) had already been treated with anthracyclines.

At time of MOC administration most patients presented with abdominal carcinomatosis only or associated with other site of disease (N = 43, 79.6%), while only 11 patients presented with isolated parenchymal or lymph node disease.

### Response to treatment

At time of analysis, all patients were assessable for response (Table 2): in the overall series, 3 complete responses (5.5%, 95% CI:-0.58, 11.6), and 8 partial responses (14.8%, 95% CI: 5.3, 24.3) have been registered, with an ORR of 20.4% (95% CI: 9.6, 31.0). Median response duration was 13 months (range = 3-35), and 8 out of 11 patients (72.7%) had a response duration ≥6 months (data not shown). Eleven patients (20.4%, 95% CI: 9.6, 31.0) experienced stable disease (median duration: 6 months, range = 3-15), and 8 of them had a response duration ≥6 months. Clinical benefit (complete and partial response plus stable disease) was achieved in 22 cases (40.7%, 95% CI: 27.6, 53.8), and 16 of them (72.7%) experienced a duration of clinical benefit ≥6 months. A total of 32 patients (59.2.%, 95% CI: 46.1, 72.3) progressed during treatment.

**Table 2:** **Clinical response in the overall series and according to platinum sensitivity**

	N.	Complete N. (%) ***CI95%***	Partial N. (%) ***CI95%***	ORR N. (%) ***CI95%***	Stable disease N. (%) ***CI95%***	Clinical benefit N. (%) ***CI95%***	PD N. (%) ***CI95%***	p value ^a^
**All cases**	**54**	**3 ( 5.5)**	**8 (14.8)**	**11 (20.4)**	**11 (20.4)**	**22 (40.7)**	**32 (59.2)**	
		*-0.58,11.6*	*5.3, 24.3*	*9.6,31.0*	*9.6,31.0*	*27.6, 53.8*	*46.1,72.3*	
**Refractory**	***20***	**1 ( 5.0)**	**2 (10.0)**	**3 (15.0)**	**6 (30.0)**	**9 (45.0)**	**11 (55.0)**	
**Resistant**		*-4.5, 14.6*	*-3.1,23.1*	*-0.6,30.6*	*9.9, 50.1*	*23.2, 66.8*	*33.2, 76.8*	
**Sensitive**	**34**	**2 ( 5.9)**	**6 (17.6)**	**8 (23.5)**	**5 (14.7)**	**13 (38.2)**	**21 (61.7)**	
		*-2.0, 13.8*	*4.8,30.4*	*9,2,37.8*	*2.8, 26.7*	*21.9, 54.5*	*45.3, 78.0*	0.5^**b**^

^a^Calculated by Fisher’s exact test for proportion.

^b^complete/partial *versus* stable disease/progression, in platinum sensitive *versus* platinum refractory/resistant disease.

In platinum refractory/resistant disease, we registered 1 complete and 2 partial responses (ORR = 15.0%; 95% CI:-0.6, 30.6); stabilization of disease was observed in 6 out of 20 patients (30.0%, 95% CI: 9.9,50.1). In platinum sensitive patients, ORR was 23.5% (95% CI: 9.2,37.8); stabilization of disease was observed in 5 out of 34 patients (14.7%, 95% CI: 2.8, 26.7).

As shown in Table 3, response according to GCIG criteria was significantly associated with RECIST defined response to treatment: in particular, return of Ca125 to normal levels, or ≥50% reduction compared to baseline predicted ORR in 56.2% of cases, whereas lack of response according to GCIG criteria predicted lack of RECIST assessed response in all cases.

**Table 3:** **Variables associated with RECIST defined response to CTX**

		Clinical response		p value ^a^
Variable	All cases N.	Complete and Partial response N. (%)	Stable and Progressive disease N. (%)	
**GCIG response**				
Complete/Partial	16	9 (56.2)	7 (43.7)	
SD/Progression	35	0	35 (100)	**0.0001** ^**b**^
**Histotype**				
Serous	45	7 (15.5)	38 (84.4)	
Other	9	4 (44.4)	5 (55.5)	**0.07**
**Pattern of recurrence**				
Carcinomatosis	43	9 (20.9)	34 (79.1)	
Isolated lymph node or parenchymal disease	11	2 (18.2)	9 (81.8)	0.69
**Platinum sensitivity**				
Refractory/resistant	20	3 (15.0)	17 (85.0)	
Sensitive	34	8 (23.5)	26 (76.5)	0.51
**No. previous chemotherapy lines**				
≤4	34	7 (20.6)	27 (79.4)	
>4	20	4 (20.0)	16 (80.0)	0.38

^a^Calculated by Fisher’s exact test for proportion, ^b^Calculated on 51 cases with baseline Ca125 levels > 35 I.U./ml.

Percentages may not total 100% due to rounding.

Patients with serous versus other tumor histotypes showed a lower rate of response to MOC compared to other tumor histotypes, although the statistical significance was not reached.

Pattern of disease progression did not influence clinical response to treatment. Finally, neither was there any difference in response rate according to initial platinum sensitivity, nor with number of previous chemotherapy lines (Table 3).

### Survival analysis

As of August 2013, follow up data were available for all patients: median follow up duration was 10 months (range: 4–43). During the follow up period, progression and death of disease were observed in 52 (96.3%), and 37 (68.5%) cases, respectively.

**Figure 1:**
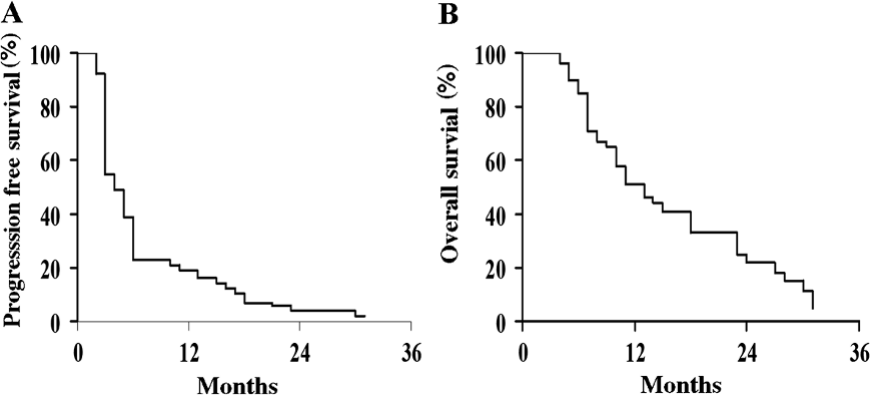
**PFS (A) and OS (B) curves in the overall series.**

**Figure 2:**
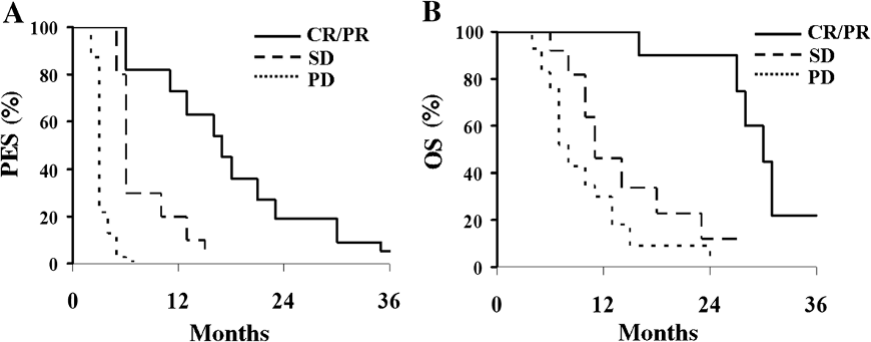
**PFS (A) and OS (B) according to response to MOC.**

In the whole series median PFS was 4 months, and the 12-month PFS rate was 19.6% (Figure 1A); median OS was 13 months, and the 12-month OS rate was 51.5% (Figure 1B). Patients achieving complete or partial response to MOC showed a more favorable PFS (median = 17 months) compared to patients with stabilization (median = 6 months; p value = 0.009) or progression of disease (median = 3 months; p value = 0.0001) (Figure 2A). Median OS of responding patients was 30 months compared to 11 months in cases achieving stabilization (p value = 0.0011), or progression of disease (median = 8 months; p value = 0.0001) (Figure 2B). There was no difference in PFS curve according to initial platinum sensitivity (data not shown).

### Toxicity

A total of 370 courses were evaluable for toxicity, with a median number of 4 cycles (range = 2-35) having been administered per patient. Cumulative dose of MOC ranged between 2,900 mg to 52,000 mg (median = 6,000 mg). Hematological toxicity was documented in only 1 patient who experienced grade 3 anemia. Non-hematological toxicity was registered in 4 patients: in particular, 1 patient experienced grade 2 nausea/vomiting and grade 3 asthenia, 1 patient showed grade 1 cutaneous toxicity, 1 patient referred grade 1 pruritus, and 1 patient had to discontinue treatment due to grade 3 gastritis.

## Discussion

This retrospective, multicenter study was aimed at investigating the efficacy and toxicity of MOC in very heavily treated ROC patients.

The very early study by Beck et al. [13], who utilized MOC in 78 advanced ovarian cancer patients no longer amenable to surgery or radiotherapy reported a response rate of 48%; however, this figure referred to patients treated during the time frame preceding the advent of platinum agents in the scenario of medical treatment of ovarian cancer. This consideration, together with the use of response evaluation criteria completely different from the current standard, intuitively precluded any reliable comparison with our results. A more recent feasibility study including 14 ROC patients documented an ORR to MOC of 7.1%, while a retrospective review of a similar patient setting reported a 44% response rate [16]; nonetheless, the reliability of these data are heavily impaired by the very small sample series [15, 16].

In this context, our study on 54 patients first allows to more reliably assess i) the extent of clinical efficacy of MOC, ii) the association between clinico-pathological features and response, and iii) the impact of sensitivity to MOC in terms of clinical outcome.

Indeed, MOC provided in our series an ORR of 20.4%, a figure which appears in the range reported for other drugs used in a similar setting [22, 23]; the achievement of an ORR of 15.0% in platinum resistant subgroup should also be not underestimated considering the corresponding figures obtained with intravenously administered cytotoxic drugs [3, 22–25]. Moreover, 20.4% of patients experienced stabilization of disease, thus leading to a rate of clinical benefit of 40.8%, which seems clinically relevant considering that almost 80% of patients had already received ≥3 treatments before MOC administration.

In addition, the documentation of ≥6 month duration of clinical benefit in about 30% of cases confirms earlier, anedoctal experiences about MOC-induced long lasting control of disease [14]; in this context, recent preclinical and clinical evidences have demonstrated that metronomic cyclophosphamide used alone or in combination with other agents is able to selectively reduce circulating immunosuppressive T-regulatory cells and myeloid derived suppressor cells, while inducing antitumor T cell response [25, 26]; moreover, MOC has been also shown to inhibit cancer stem cells *in vitro* and *in vivo* [27, 28], thus suggesting that MOC could act as a multi-targeted approach [11, 12].

As far as clinical outcome is concerned, median PFS and OS were comparable to those achieved with other cytotoxic agents in retrospective as well as phase II studies in heavily treated ROC [22–24]; moreover, patients responding to MOC experienced a very favorable PFS (median = 17 months), and OS (median = 30 months) compared to non-responders. It remains to be clarified why survival curves as well as tumor responsiveness did not show any difference according to platinum sensitivity: it is conceivable that, given the not negligible number of chemotherapy lines preceding the administration of MOC, the potential favorable implications of “platinum-sensitivity” of disease as defined on the basis of initial platinum-free interval could be less evident; alternatively, it cannot be excluded that the relatively low number of platinum resistant/refractory patients could have precluded the detection of a statistically significant difference in clinical outcome between groups.

Finally, it has also to be acknowledged that the effects of metronomic chemotherapy administration on tumor microenvironment could sustain the independence of MOC activity from tumor cell sensitivity to the drug [11, 12].

As far as the toxicity is concerned, continuously administered MOC at the dose of 50 mg daily appeared to be well tolerated despite the number of previously administered chemotherapy lines: indeed, grade 3 toxicity was documented in 3 patients, and only 1 of them required treatment discontinuation. This profile of toxicity thus appears more favorable compared to previously reported experiences with MOC which, nonetheless, utilized slightly higher doses of the drug, i.e. 50–150 mg daily [13], or 50 mg twice a day for 3 weeks with a 1-week break [15], or 50–150 mg d1-14, every 4 weeks [16]. This observation, together with the recognized anti-angiogenic activity of MOC have provided the rationale for its combination with bevacizumab in retrospective as well as prospective studies which showed values of median PFS (range = 3.0-7.2 months) [29–31] comparable to our and previous results [16]. However, with the limits inherent to the direct comparison of different samples and studies, the combination MOC plus bevacizumab appeared endowed with a higher percentage of treatment related adverse events [29–32], thus highlighting the potential role of MOC as a valid alternative in terms of risk/benefit ratio.

In this context, the combination of MOC with other anti-angiogenic drugs, such as nindetanib (NCT01610869) or pazopanib (NCT01238770) (http://www.clinicaltrials.gov) seems quite appealing. Finally, future therapeutic development for MOC could be represented by combination with immunotherapeutic approaches [11, 12, 33].

## Conclusion

Metronomic oral administration of cyclophosphamide single agent seems to provide a valid alternative in terms of risk/benefit ratio in the scenario of palliative treatments of heavily treated ROC patients, especially if affected by several comorbidities.
